# A Cross-Sectional Study to Assess Diastolic Function in Young Adults With Type 2 Diabetes Mellitus

**DOI:** 10.7759/cureus.111458

**Published:** 2026-06-24

**Authors:** Abhishek Das, Aashish Arumugam, Surendar P B, Magesh Vadivelu

**Affiliations:** 1 Cardiology, Meenakshi Medical College Hospital and Research Institute, Meenakshi Academy of Higher Education and Research, Kanchipuram, IND

**Keywords:** diabetic cardiomyopathy, diastolic dysfunction, echocardiography, type 2 diabetes mellitus, young adults

## Abstract

Introduction: Type 2 diabetes mellitus (T2DM) is increasingly prevalent among young adults and is associated with early cardiovascular complications, including diabetic cardiomyopathy and left ventricular diastolic dysfunction. Early identification of subclinical cardiac dysfunction may facilitate risk stratification and clinical evaluation of cardiovascular involvement. The present study aimed to assess left ventricular diastolic function and its association with glycemic control and duration of diabetes in young adults with T2DM using echocardiography.

Materials and methods: This hospital-based cross-sectional observational study was conducted in the Department of Cardiology at Meenakshi Medical College Hospital and Research Institute, Kanchipuram, Tamil Nadu, over a period of 12 months. A total of 150 young adults aged less than 40 years with T2DM were enrolled. Detailed clinical evaluation, biochemical investigations, and two-dimensional transthoracic echocardiography were performed in all participants. Echocardiographic assessment included left ventricular mass, interventricular septal thickness, E/A ratio, E/e’ ratio, and ejection fraction. Left ventricular diastolic dysfunction was graded according to available standard echocardiographic criteria.

Results: The mean age of the study population was 34.8 ± 4.2 years, with males constituting 88 (58.7%) participants. Left ventricular diastolic dysfunction was observed in 68 (45.3%) participants, with Grade I dysfunction being the most common abnormality (48 (32.0%)). The mean E/A ratio was reduced (1.02 ± 0.28), while the E/e’ ratio was elevated (11.2 ± 2.6), suggestive of impaired diastolic function. Significant associations were observed between diastolic dysfunction and poor glycemic control (χ²=16.84, p<0.001) and longer duration of diabetes (χ²=12.31, p=0.002).

Conclusion: Young adults with T2DM demonstrated a high frequency of subclinical left ventricular diastolic dysfunction despite preserved systolic function. Poor glycemic control and longer duration of diabetes were significantly associated with diastolic dysfunction. Given the cross-sectional design of the study, these findings should be interpreted as associations rather than causal relationships. Further prospective studies are required to determine the clinical significance and prognostic implications of these findings.

## Introduction

Type 2 diabetes mellitus (T2DM) has emerged as one of the most significant global public health challenges, with rapidly increasing prevalence among younger populations due to sedentary lifestyle, obesity, unhealthy dietary habits, and urbanization [1,2]. Earlier onset of T2DM in young adults results in prolonged exposure to hyperglycemia and metabolic disturbances, thereby increasing the risk of early cardiovascular complications and premature morbidity [2]. Cardiovascular disease remains the leading cause of mortality among patients with diabetes mellitus, accounting for a substantial proportion of long-term complications associated with the disease [3].

Apart from accelerating atherosclerosis and coronary artery disease, diabetes mellitus can directly affect myocardial structure and function, leading to diabetic cardiomyopathy [4]. Diabetic cardiomyopathy is characterized by ventricular dysfunction occurring independently of hypertension, valvular heart disease, or ischemic heart disease. Left ventricular diastolic dysfunction is considered one of the earliest manifestations of diabetic cardiomyopathy and often precedes the development of overt systolic dysfunction and symptomatic heart failure [5]. Furthermore, heart failure with preserved ejection fraction has been increasingly linked to T2DM, with impaired ventricular relaxation and myocardial stiffness playing a central role in its pathogenesis [6].

Early stages of diastolic dysfunction are frequently asymptomatic and therefore remain clinically undetected unless actively evaluated [7]. Persistent hyperglycemia, oxidative stress, myocardial fibrosis, insulin resistance, and accumulation of advanced glycation end products contribute significantly to structural and functional myocardial abnormalities in individuals with diabetes [4,8]. Several studies have demonstrated increased left ventricular mass, altered ventricular geometry, and impaired diastolic function among patients with T2DM [9,10]. Recent evidence also suggests that younger patients with T2DM may develop early subclinical myocardial dysfunction despite preserved systolic function and absence of overt cardiovascular symptoms [11].

Echocardiography is a simple, non-invasive, and reliable imaging modality widely used for the assessment of cardiac structure and ventricular function [7]. Doppler echocardiography and tissue Doppler imaging play important roles in detecting early abnormalities in left ventricular diastolic function and in identifying subclinical diabetic cardiomyopathy before the onset of symptomatic heart failure [12]. Early identification of cardiac dysfunction in patients with diabetes may facilitate timely intervention, optimization of glycemic control, and aggressive management of cardiovascular risk factors, thereby reducing future cardiovascular morbidity and mortality [13].

Young adults with T2DM constitute a particularly high-risk group because cardiovascular abnormalities may develop early during the course of the disease and remain undetected for prolonged periods. India is currently home to more than 100 million individuals with diabetes, with a rapidly increasing burden among younger adults, making early identification of cardiovascular complications a major public health priority. Recent national estimates indicate that the prevalence of diabetes and prediabetes continues to rise across both urban and rural populations in India. Despite this growing burden, data evaluating subclinical left ventricular diastolic dysfunction specifically in young adults with T2DM remain scarce. Most available studies have included mixed age groups, focused on older adults, or have not specifically examined the relationship between diastolic dysfunction, glycemic control, and duration of diabetes in young Indian patients. Therefore, the present study aimed to evaluate left ventricular diastolic function using echocardiography and to examine its association with glycemic control and duration of diabetes among young adults with T2DM.

## Materials and methods

This hospital-based cross-sectional observational study was conducted in the Department of Cardiology at Meenakshi Medical College Hospital and Research Institute, Kanchipuram, Tamil Nadu, over a period of 12 months, from January 2025 to December 2025. The study included young adults aged less than 40 years diagnosed with T2DM who attended the outpatient and inpatient departments of Cardiology and General Medicine during the study period. Eligible participants were recruited using a consecutive sampling method until the required sample size was achieved. Participants were enrolled after obtaining written informed consent. Institutional Ethics Committee approval was obtained before the commencement of the study (MMCHRI-IEC/PG/05/Nov24).

The sample size was calculated using the standard formula for cross-sectional studies, n = Z²pq/d², where “n” represents the required sample size, “Z” is the standard normal variate corresponding to a 95% confidence interval (1.96), “p” is the prevalence of left ventricular diastolic dysfunction, “q” is calculated as 1−p, and “d” is the allowable error. Based on the study conducted by Chaudhary et al., the prevalence of left ventricular diastolic dysfunction among patients with T2DM was 41% [12]. Taking p=0.41, q=0.59, and allowable error as 20% of the prevalence, the calculated minimum sample size was approximately 138 participants. After accounting for possible non-response and incomplete data, the final sample size was rounded off to 150 participants.

The inclusion and exclusion criteria used for participant selection are presented in Table 1.

**Table 1:** Inclusion and exclusion criteria

Inclusion criteria	Exclusion criteria
Patients diagnosed with type 2 diabetes mellitus	Patients with type 1 diabetes mellitus
Age less than 40 years	Patients with ischemic heart disease
Either gender	Patients with valvular heart disease
Willingness to participate in the study	Patients with congenital heart disease
	Patients with chronic kidney disease
	Patients with chronic liver disease
	Pregnant women
	Patients with cardiomyopathy of other etiologies

Detailed clinical history, including duration of diabetes, smoking status, and associated comorbidities, was obtained from all participants. All study participants underwent biochemical investigations, including fasting blood sugar, postprandial blood sugar, glycated hemoglobin (HbA1c), lipid profile, and renal function tests. For analytical purposes, HbA1c levels were categorized into three groups as <7%, 7%-9%, and >9% to assess the association between glycemic control and left ventricular diastolic dysfunction. These categories were selected to represent good glycemic control (<7%), suboptimal control (7%-9%), and poor glycemic control (>9%) based on commonly accepted clinical practice recommendations.

Two-dimensional transthoracic echocardiography was performed in all participants using a Vivid T8 echocardiographic system (GE Healthcare, Chicago, Illinois, USA) in the Department of Cardiology by two experienced cardiologists to minimize inter-observer variability and ensure accuracy of measurements. Echocardiographic examinations were performed with participants in the left lateral decubitus position according to standard American Society of Echocardiography (ASE) recommendations. Measurements were obtained from parasternal long-axis, parasternal short-axis, and apical four-chamber views, and the average of three consecutive cardiac cycles was used for analysis whenever feasible. The cardiologists performing image interpretation were blinded to participants’ biochemical parameters, including HbA1c values.

Echocardiographic parameters assessing cardiac structure included left ventricular mass, interventricular septal thickness, posterior wall thickness, and left atrial diameter. Left ventricular diastolic function was evaluated using Doppler echocardiographic parameters, including mitral inflow E-wave velocity, A-wave velocity, E/A ratio, tissue Doppler-derived e′ velocity, and E/e′ ratio. Left ventricular ejection fraction was assessed to evaluate systolic function.

Diastolic dysfunction was assessed using a simplified approach based on available ASE/European Association of Cardiovascular Imaging (EACVI)-recommended Doppler and tissue Doppler parameters, including E/A ratio, E/e′ ratio, e′ velocity, and left atrial size. Tricuspid regurgitation velocity and left atrial volume index were not routinely available and therefore were not included in the assessment. Accordingly, the classification of left ventricular diastolic dysfunction should be interpreted as a simplified echocardiographic assessment rather than a complete application of the 2016 ASE/EACVI diagnostic algorithm [7]. To reduce measurement variability, all studies were independently reviewed by two experienced cardiologists, and any discrepancies were resolved by consensus.

Data were entered into Microsoft Excel (Microsoft Corp., Redmond, WA, USA) and analyzed using Statistical Package for Social Sciences (SPSS) software version 26.0 (IBM Corp., Armonk, NY, USA). Continuous variables were expressed as mean ± standard deviation, while categorical variables were presented as frequency and percentage. Associations between categorical variables were analyzed using the Chi-square test. As the primary objective of the study was a descriptive assessment of diastolic dysfunction prevalence and its unadjusted associations with selected clinical variables, multivariable regression analysis was not performed. A p-value of less than 0.05 was considered statistically significant.

## Results

The demographic profile of the study participants showed that the mean age was 34.8 ± 4.2 years. Male participants constituted 58.7% (88) of the study population, while females accounted for 41.3% (62). The mean duration of T2DM was 6.4 ± 3.1 years, and the mean body mass index was 28.6 ± 4.5 kg/m², indicating an overweight population. Additionally, 36 (24.0%) participants were smokers and 54 (36.0%) had hypertension, indicating the presence of several potential cardiovascular risk factors that may influence cardiac function in this study population (Table 2).

**Table 2:** Demographic characteristics of study participants (n=150) Data are presented as mean ± SD or n (%). BMI = Body Mass Index; SD = Standard Deviation.

Variable	Value
Age (years)	34.8 ± 4.2
Male	88 (58.7%)
Female	62 (41.3%)
Duration of diabetes (years)	6.4 ± 3.1
BMI (kg/m²)	28.6 ± 4.5
Smokers	36 (24.0%)
Hypertension	54 (36.0%)

The biochemical profile of the participants demonstrated poor glycemic control, with mean fasting blood sugar, postprandial blood sugar, and HbA1c levels of 168.4 ± 42.5 mg/dL, 248.7 ± 58.3 mg/dL, and 8.6 ± 1.4%, respectively. Lipid profile analysis revealed elevated total cholesterol, low-density lipoprotein cholesterol, and triglyceride levels, while high-density lipoprotein cholesterol levels were relatively lower. The mean systolic and diastolic blood pressure values were also elevated, demonstrating the coexistence of multiple cardiovascular risk factors within the study population (Table 3).

**Table 3:** Clinical and biochemical parameters of study participants Data are presented as mean ± SD. HbA1c = Glycated Hemoglobin; HDL = High-Density Lipoprotein; LDL = Low-Density Lipoprotein; SD = Standard Deviation.

Parameter	Mean ± SD
Fasting blood sugar (mg/dL)	168.4 ± 42.5
Postprandial blood sugar (mg/dL)	248.7 ± 58.3
HbA1c (%)	8.6 ± 1.4
Total cholesterol (mg/dL)	204.3 ± 38.6
LDL cholesterol (mg/dL)	126.5 ± 31.2
HDL cholesterol (mg/dL)	41.8 ± 8.4
Triglycerides (mg/dL)	186.2 ± 46.7
Systolic blood pressure (mmHg)	132.4 ± 14.5
Diastolic blood pressure (mmHg)	84.6 ± 9.8

Echocardiographic assessment revealed structural and functional cardiac alterations among the study participants. The mean left ventricular mass was 172.5 ± 28.4 g, while the mean left atrial diameter was 36.8 ± 4.2 mm. Doppler parameters were suggestive of impaired diastolic function, with a mean E/A ratio of 1.02 ± 0.28 and a mean E/e’ ratio of 11.2 ± 2.6. However, left ventricular systolic function remained preserved, with a mean ejection fraction of 58.6 ± 5.4% (Table 4).

**Table 4:** Echocardiographic parameters of study participants Data are presented as mean ± SD. E/A Ratio = Ratio of Early (E) to Late (A) Ventricular Filling Velocity; E/e’ Ratio = Ratio of Early Mitral Inflow Velocity to Early Diastolic Mitral Annular Velocity; SD = Standard Deviation.

Echocardiographic parameter	Mean ± SD
Left ventricular mass (g)	172.5 ± 28.4
Left atrial diameter (mm)	36.8 ± 4.2
Interventricular septal thickness (mm)	10.4 ± 1.3
Posterior wall thickness (mm)	10.1 ± 1.2
Ejection fraction (%)	58.6 ± 5.4
E-wave velocity (cm/s)	72.5 ± 12.8
A-wave velocity (cm/s)	68.4 ± 10.6
E/A ratio	1.02 ± 0.28
E/e’ ratio	11.2 ± 2.6

Assessment of left ventricular diastolic function showed that 68 (45.3%) participants had evidence of diastolic dysfunction, whereas 82 (54.7%) had normal diastolic function. Grade I diastolic dysfunction was the most common abnormality observed in 48 (32.0%) participants, followed by Grade II dysfunction in 16 (10.7%) and Grade III dysfunction in 4 (2.6%) participants. These findings demonstrate a relatively high frequency of echocardiographic evidence of diastolic dysfunction within this cohort of young adults with T2DM. However, in the absence of a non-diabetic control group, comparisons with the general population should be interpreted cautiously (Table 5).

**Table 5:** Prevalence of left ventricular diastolic dysfunction among study participants (n=150) Data are presented as n (%).

Diastolic function status	n (%)
Normal diastolic function	82 (54.7%)
Grade I diastolic dysfunction	48 (32.0%)
Grade II diastolic dysfunction	16 (10.7%)
Grade III diastolic dysfunction	4 (2.6%)

A statistically significant association was observed between HbA1c levels and left ventricular diastolic dysfunction (χ²=16.84, p<0.001). Participants with HbA1c levels >9% demonstrated the highest prevalence of diastolic dysfunction (24 (66.7%)), whereas those with HbA1c levels <7% predominantly had normal diastolic function (32 (76.2%)). The findings demonstrate a significant unadjusted association between higher HbA1c levels and diastolic dysfunction. However, because multivariable adjustment for potential confounders such as hypertension, body mass index, smoking status, and dyslipidemia was not performed, these associations should be interpreted with caution (Table 6).

**Table 6:** Association between HbA1c levels and diastolic dysfunction Data are presented as n (%). Association between categorical variables was analyzed using the chi-square test. p-value <0.05 was considered statistically significant. df = Degrees of Freedom; HbA1c = Glycated Hemoglobin; χ² = Chi-Square Value.

HbA1c (%)	Normal diastolic function	Diastolic dysfunction	χ² value	df	p-value
<7%	32 (76.2%)	10 (23.8%)	16.84	2	<0.001
7%-9%	38 (52.8%)	34 (47.2%)			
>9%	12 (33.3%)	24 (66.7%)			

The prevalence of left ventricular diastolic dysfunction increased significantly with longer duration of diabetes (χ²=12.31, p=0.002). Among participants with diabetes duration greater than 10 years, 22 (64.7%) had diastolic dysfunction, compared with only 18 (31.0%) among those with duration less than 5 years. These findings demonstrate a significant association between longer duration of diabetes and the presence of diastolic dysfunction. However, the cross-sectional design of the study does not permit conclusions regarding causality or disease progression (Table 7). Exploratory analysis showed that participants with diastolic dysfunction also had a higher prevalence of hypertension, overweight/obesity, and adverse metabolic profiles. As these factors are recognized contributors to diastolic dysfunction, their potential confounding influence should be considered when interpreting the observed associations between HbA1c levels, duration of diabetes, and left ventricular diastolic dysfunction.

**Table 7:** Association between duration of diabetes and diastolic dysfunction Data are presented as n (%). Association between categorical variables was analyzed using the chi-square test. p-value <0.05 was considered statistically significant. df = Degrees of Freedom; χ² = Chi-Square Value.

Duration of diabetes	Normal diastolic function	Diastolic dysfunction	χ² value	df	p-value
<5 years	40 (69.0%)	18 (31.0%)	12.31	2	0.002
5-10 years	30 (51.7%)	28 (48.3%)			
>10 years	12 (35.3%)	22 (64.7%)			

## Discussion

The present study evaluated left ventricular diastolic function in young adults with T2DM using echocardiography. The findings demonstrated a relatively high frequency of echocardiographic evidence of subclinical left ventricular diastolic dysfunction despite preserved systolic function among young adults with T2DM. These observations are consistent with previous reports describing subclinical myocardial abnormalities in patients with diabetes mellitus. However, owing to the cross-sectional design and absence of a non-diabetic control group, the present study cannot establish whether the observed abnormalities are exclusively attributable to diabetes or determine their temporal progression.

The mean age of the study participants was 34.8 ± 4.2 years, with males constituting 58.7% (88) of the study population. These findings highlight that cardiovascular abnormalities can occur even in younger diabetic populations. Similar observations were reported by Lascar et al., who emphasized the increasing burden of early cardiovascular complications among young adults with T2DM [11]. Early onset of diabetes is clinically important because prolonged exposure to hyperglycemia and associated metabolic disturbances predisposes individuals to premature cardiovascular morbidity and mortality.

In the present study, 68 (45.3%) participants demonstrated evidence of left ventricular diastolic dysfunction, with Grade I dysfunction being the most common abnormality observed in 48 (32.0%) participants. Grade II and Grade III dysfunction were identified in 16 (10.7%) and 4 (2.6%) participants, respectively. Comparable findings were reported by Chaudhary et al., who observed diastolic dysfunction in nearly 41 (41%) patients with T2DM and demonstrated a significant association with poor glycemic control and longer duration of diabetes [12]. The slightly higher prevalence observed in the present study may reflect differences in participant characteristics, including glycemic status, duration of diabetes, and coexistence of cardiovascular risk factors. However, these possibilities cannot be confirmed because multivariable adjustment was not performed.

Echocardiographic evaluation revealed impaired ventricular relaxation, as evidenced by a reduced E/A ratio (1.02 ± 0.28) and an elevated E/e’ ratio (11.2 ± 2.6). Increased left ventricular mass and mild alterations in ventricular wall thickness were also observed. Similar findings were demonstrated by Tadic et al., who reported significantly impaired diastolic parameters among patients with diabetes compared with healthy controls [14]. These abnormalities are consistent with echocardiographic patterns reported in patients with diastolic dysfunction and diabetic cardiomyopathy. However, because the assessment was based on a simplified echocardiographic approach without routine measurement of tricuspid regurgitation velocity and left atrial volume index, the findings should be interpreted within the context of these methodological limitations.

Despite evidence of significant diastolic dysfunction, left ventricular systolic function remained preserved in the present study, with a mean ejection fraction of 58.6 ± 5.4%. This finding is consistent with the observations of Jiang et al., who demonstrated that diabetic cardiomyopathy predominantly affects diastolic function during the early stages, before progression to overt systolic dysfunction [15]. Preservation of systolic function in asymptomatic individuals further supports the importance of echocardiography in the early detection of subclinical myocardial abnormalities in patients with diabetes.

A statistically significant association was observed between poor glycemic control and left ventricular diastolic dysfunction (p<0.001). Participants with HbA1c levels >9% demonstrated markedly higher prevalence of diastolic dysfunction than those with HbA1c <7%. Similar findings were reported by Chaudhary et al., who demonstrated a strong positive association between elevated HbA1c levels and severity of diastolic dysfunction [12]. Previous studies have suggested that chronic hyperglycemia may contribute to myocardial fibrosis, oxidative stress, endothelial dysfunction, and accumulation of advanced glycation end products, which may adversely affect myocardial relaxation and ventricular compliance [16]. Nevertheless, because the present analysis was unadjusted, the observed association between HbA1c levels and diastolic dysfunction should not be interpreted as evidence of an independent causal relationship.

The present study also demonstrated a significant association between duration of diabetes and diastolic dysfunction (p=0.002). Although a significant association was observed, causality cannot be inferred from the present cross-sectional study. Furthermore, factors such as age, hypertension, obesity, smoking status, dyslipidemia, and medication use may have influenced the observed relationship. Participants with diabetes duration greater than 10 years showed substantially higher prevalence of cardiac dysfunction than those with shorter duration of disease. Similar observations were reported in the systematic review by Palanisamy et al., who concluded that both the prevalence and severity of diastolic dysfunction progressively increase with longer duration of diabetes mellitus [17]. Increased body mass index, hypertension, and dyslipidemia were also commonly observed among participants with diastolic dysfunction. Ceriello et al. similarly reported that obesity, hypertension, insulin resistance, and dyslipidemia accelerate myocardial structural changes and increase the risk of heart failure in patients with T2DM [18].

Recent evidence further supports the high prevalence of diabetic cardiomyopathy among patients with T2DM. Swiatkiewicz et al. reported that a substantial proportion of patients with diabetes had evidence of left ventricular diastolic dysfunction despite the absence of overt symptoms [19]. Similarly, Chen et al. emphasized that more than 40% of individuals with T2DM may exhibit subclinical diastolic dysfunction even in the absence of systolic impairment [20]. These findings are comparable to the results of the present study and suggest that subclinical diastolic abnormalities are frequently identified in patients with T2DM. Further prospective studies incorporating control groups and comprehensive multivariable analyses are required to determine the clinical significance of these findings and the potential role of screening strategies.

Strengths and limitations

Strengths of the present study include its focus on young adults with T2DM, a relatively understudied but clinically important population at risk for early cardiovascular complications. Comprehensive echocardiographic assessment using Doppler and tissue Doppler imaging enabled evaluation of subclinical cardiac abnormalities in asymptomatic individuals.

However, several limitations should be considered while interpreting the findings. First, the cross-sectional design precludes determination of temporal or causal relationships between glycemic control, duration of diabetes, and diastolic dysfunction. Second, the absence of an age- and sex-matched non-diabetic control group limits comparison with the general population and prevents assessment of the extent to which the observed findings are specifically attributable to T2DM. Third, multivariable adjustment for potential confounders such as hypertension, obesity, dyslipidemia, smoking status, and medication use was not performed; therefore, the reported associations should be interpreted as unadjusted findings. Fourth, assessment of diastolic dysfunction was based on available Doppler and tissue Doppler parameters and did not routinely include tricuspid regurgitation velocity or left atrial volume index, which are components of the complete ASE/EACVI 2016 diagnostic algorithm. Finally, the study was conducted at a single tertiary care center with a moderate sample size and without longitudinal follow-up, which may limit generalizability and preclude evaluation of long-term cardiovascular outcomes.

Future multicentric prospective studies incorporating healthy control groups, comprehensive echocardiographic assessment, multivariable analyses, and long-term follow-up are warranted to better define the determinants and prognostic significance of subclinical diastolic dysfunction in young adults with T2DM.

## Conclusions

The present study demonstrated a relatively high frequency of echocardiographic evidence of subclinical left ventricular diastolic dysfunction among young adults with T2DM despite preserved systolic function. Echocardiographic abnormalities were significantly associated with poor glycemic control and longer duration of diabetes. However, given the cross-sectional design and absence of multivariable adjustment, these findings should be interpreted as unadjusted associations rather than evidence of independent causal relationships.

The findings suggest that subclinical cardiac abnormalities may be present in a substantial proportion of young adults with T2DM and highlight the potential utility of echocardiographic assessment in identifying such abnormalities. Further prospective studies incorporating healthy control groups, comprehensive echocardiographic evaluation, and longitudinal follow-up are required to clarify the determinants, clinical significance, and prognostic implications of left ventricular diastolic dysfunction in this population.
